# Supplementary material for: Dynamics of Physicochemical Properties, Functional Compounds and Antioxidant Capacity during Spontaneous Fermentation of Lycium ruthenicum Murr. (Qinghai–Tibet Plateau) Natural Vinegar
Source: Foods. 2022 May 5;11(9):1344. doi: 10.3390/foods11091344 (PMC9104459; doi:10.3390/foods11091344)
Supplement: Supplementary file 1 [file foods-11-01344-s001.zip › foods-1698412-supplementary.pdf]

foods

MDPI

Supplementary

Dynamics of physicochemical Properties, Functional Compounds and Antioxidant Capacity during Spontaneous Fermentation of *Lycium Ruthenicum* Murr. (Qinghai–Tibet Plateau) Natural Vinegar

Qingchao Gao [1,2†], Yangbo Song [1,†], Ying Liang [2], Yahui Li [2], Yingjiu Chang [1], Rong Ma [1], Xiaohai Cao [1,4] and Shulin Wang [1,3,4,*]

[1] College of Agriculture and Animal Husbandry, Qinghai University, Xining, 810016, China;
[2] Key Laboratory of Food Quality and Safety of Jiangsu Province, Institute of Food Safety and Nutrition, Jiangsu Academy of Agricultural Science, Nanjing, 210014, China;
[3] State Key Laboratory of Plateau Ecology and Agriculture, Qinghai University, Xining, 810016, China;
[4] United Laboratory of Food Research and Testing of Qinghai-Gansu Province, Xining, 810016, China;
† These authors contributed equally to this work.
* Correspondence: Shulin Wang, E-mail: 1992990010@qhu.edu.cn

Table S1. Calibration curve results of 13 organic acids.

Organic Acid	Peak Order	Keep Time/Min	Regression Equation	Correlation Coefficient(R^2)
GABA	1	3.947	$y = 0.5291x-0.0016$	1.0000
Tartaric acid	2	5.250	$y = 2.2249x-0.0808$	0.9999
Citric acid	3	5.427	$y = 69.3621x-0.1281$	1.0000
Malic acid	4	5.570	$y = 1.1467x+0.0124$	1.0000
Shikimic acid	5	6.080	$y = 23.6795x+0.1988$	0.9999
Lactic acid	6	6.350	$y = 0.8297x+0.0610$	1.0000
Acetic acid	7	6.960	$y = 0.8060x+0.2466$	1.0000
Oxalic acid	8	7.430	$y = 1.6418x+0.0204$	1.0000
Succinic acid	9	7.897	$y = 0.8693x+0.0881$	1.0000
Ascorbic acid	10	9.633	$y = 0.1284x+0.0051$	1.0000
Sorbic acid	11	10.873	$y = 17.2447x-2.2395$	0.9992
Fumaric acid	12	11.547	$y = 108.6913x+0.9134$	0.9998
Gallic acid	13	13.830	$y = 147.2094x+0.2695$	1.0000

Table S2. The dynamics of metabolites and DPPH radical scavenging activity (DPPH) during BW fermentation.

Index	0 d	5 d	10 d	15 d	20 d	25 d	30 d	35 d	40 d	45 d	50 d	55 d	60 d
Total acid (mg/mL)	4.03 ± 0.92g	10.03 ± 1.82f	9.95 ± 0.87f	17.71 ± 1.53d	15.28 ± 0.42e	18.92 ± 2.66cd	25.17 ± 0.9a	20.55 ± 2.03bc	21.91 ± 0.73b	21.16 ± 0.1b	19.69 ± 0.79c	22.21 ± 1.67b	18.21 ± 0.97cd
Total sugar (mg/mL)	109.76 ± 11.55a	52.64 ± 6.19b	30.81 ± 1.93c	7.77 ± 1.31d	2.8 ± 0.06e	3.09 ± 0.12e	3.11 ± 0.14e	3.42 ± 0.12e	3.38 ± 0.08e	3.5 ± 0.17e	3.37 ± 0.07e	3.38 ± 0.03e	3.72 ± 0.15e
Reducing sugar (mg/mL)	57.81 ± 0.26a	19.02 ± 0.04b	10.46 ± 0.05c	3.32 ± 0.25d	0.94 ± 0.02e	1.21 ± 0.03e	0.83 ± 0.02e	0.93 ± 0.04e	1.03 ± 0.02e	1.22 ± 0.03e	1.13 ± 0.02e	1.03 ± 0.01e	1.24 ± 0.10e
Total anthocyanins (mg/mL)	0.71 ± 0.01ab	0.74 ± 0.01a	0.69 ± 0.01bc	0.69 ± 0.01bc	0.68 ± 0.01bc	0.66 ± 0.01c	0.43 ± 0.01f	0.44 ± 0.01ef	0.47 ± 0.01e	0.69 ± 0.02bc	0.68 ± 0.01bc	0.62 ± 0.01d	0.67 ± 0.01c
Total flavonoid (mg/mL)	0.97 ± 0.06g	2.52 ± 0.23e	2.81 ± 0.17de	3.33 ± 0.04cd	2.66 ± 0.05de	2.08 ± 0.05f	3.44 ± 0.01c	3.33 ± 0.04c	3.5 ± 0.04c	3.66 ± 0.06b	3.39 ± 0.02c	3.56 ± 0.03b	3.99 ± 0.06a
Total polyphenol (mg/mL)	2.75 ± 0.01d	2.95 ± 0.01d	2.86 ± 0.02d	3.19 ± 0.04cd	2.74 ± 0.01d	2.3 ± 0.03e	3.31 ± 0.01c	3.58 ± 0.03b	3.57 ± 0.01b	3.7 ± 0.02b	3.62 ± 0.03b	3.73 ± 0.02b	3.93 ± 0.02a
Total protein (mg/mL)	0.51 ± 0.05ce	0.55 ± 0.06bc	1.37 ± 0.03a	0.63 ± 0.03b	0.5 ± 0.04ce	0.41 ± 0.03ef	0.46 ± 0.06cef	0.36 ± 0.04fg	0.39 ± 0.07f	0.48 ± 0.01ce	0.26 ± 0.04g	0.27 ± 0.06g	0.37 ± 0.04f
SOD (mg/mL)	514.65 ± 4.27g	1721.61 ± 70.81a	1725.46 ± 21.79a	1188.82 ± 62.26b	1168.28 ± 95.69b	1104.09 ± 51.29b	607.3 ± 86.88e	575.08 ± 69.79f	565 ± 38.46f	664.71 ± 29.91d	648.09 ± 10.68d	642.05 ± 14.96d	754.35 ± 18.46c

Amylase (mg/mL)	157.38 ± 11.46c	137.03 ± 10.66d	113.9 ± 19.65fg	104.97 ± 5.4g	125.02 ± 6.31ef	131.46 ± 15.13de	158.99 ± 27.01c	104.38 ± 12.62g	115.65 ± 26.69f	169.53 ± 9.3b	169.82 ± 19.36b	207.01 ± 15.12a	196.46 ± 16.9a
DPPH (%)	29.62 ± 3.64f	46.86 ± 1.66e	62.47 ± 2.31d	66.5 ± 3.21bc	68.31 ± 2ab	66.63 ± 1.11bc	67.11 ± 0.71b	68.85 ± 0.37ab	68.57 ± 0.28ab	64.23 ± 1.48cd	66.06 ± 0.68bc	70.29 ± 2.07a	63.93 ± 3.21d

Different letters in each row mean significant differences ($p < 0.05$). Samples were collected at 0, 5th, 10th, 15th, 20th, 25th, 30th, 35th, 40th, 45th, 50th, 55th and 60th day, respectively.

Table S3. The contents of organic acids during BW fermentation (mg/mL).

organic acid	0 d	5 d	10 d	15 d	20 d	25 d	30 d	35 d	40 d	45 d	50 d	55 d	60 d
Gallic acid	0.001 ± 0.000f	0.002 ± 0.000f	0.008 ± 0.000c	0.005 ± 0.000de	0.001 ± 0.000f	0.004 ± 0.000e	0.005 ± 0.000de	0.007 ± 0.000cd	0.009 ± 0.000c	0.007 ± 0.000cd	0.014 ± 0.001b	0.015 ± 0.001ab	0.017 ± 0.001a
GABA	0.387 ± 0.009d	1.130 ± 0.004c	1.434 ± 0.008ab	1.314 ± 0.010b	0.997 ± 0.010c	1.685 ± 0.009a	1.641 ± 0.003a	1.390 ± 0.003b	1.459 ± 0.008a	1.029 ± 0.009c	1.411 ± 0.007ab	1.570 ± 0.012a	1.675 ± 0.001a
Oxalic acid	0.844 ± 0.000a	0.156 ± 0.000d	0.130 ± 0.001e	0.161 ± 0.001d	0.137 ± 0.000e	0.327 ± 0.001b	0.277 ± 0.000c	0.323 ± 0.000b	0.372 ± 0.002b	0.267 ± 0.000c	0.106 ± 0.000f	0.138 ± 0.000e	0.249 ± 0.000c
Citric acid	0.011 ± 0.000a	0.012 ± 0.000a	0.011 ± 0.000a	0.011 ± 0.000a	0.010 ± 0.000a	0.014 ± 0.001a	0.009 ± 0.000a	0.008 ± 0.000a	0.009 ± 0.001a	0.010 ± 0.000a	0.005 ± 0.001a	0.010 ± 0.001a	0.012 ± 0.000a
Succinic acid	0.760 ± 0.002	-	-	-	-	-	-	-	-	-	-	-	-
Acetic acid	-	0.063 ± 0.000d	0.253 ± 0.001a	0.252 ± 0.000a	0.041 ± 0.000e	0.171 ± 0.001b	0.096 ± 0.000c	0.007 ± 0.000f	-	-	-	-	0.173 ± 0.000b
Lactic acid	-	18.887 ± 0.003d	10.279 ± 0.000f	15.681 ± 0.001e	19.982 ± 0.001cd	22.208 ± 0.000ab	21.793 ± 0.001bc	23.147 ± 0.001a	23.415 ± 0.000a	16.622 ± 0.001e	22.692 ± 0.000a	23.427 ± 0.001a	24.314 ± 0.001a
Malic acid	0.460 ± 0.001b	0.014 ± 0.000j	0.046 ± 0.001h	0.030 ± 0.000i	0.018 ± 0.000j	0.037 ± 0.000hi	0.063 ± 0.000g	0.091 ± 0.000e	0.075 ± 0.001f	0.103 ± 0.000d	0.063 ± 0.000g	0.140 ± 0.000c	0.527 ± 0.002a
Ascorbic acid	-	0.006 ± 0.000h	0.598 ± 0.000f	-	0.810 ± 0.000e	0.810 ± 0.000e	-	0.037 ± 0.000g	-	2.213 ± 0.000d	3.476 ± 0.001b	5.170 ± 0.000a	3.390 ± 0.000c
Sorbic acid	0.384 ± 0.001a	0.388 ± 0.001a	0.417 ± 0.000a	0.398 ± 0.000a	0.401 ± 0.000a	0.394 ± 0.000a	0.378 ± 0.000a	0.372 ± 0.001a	0.379 ± 0.001a	0.377 ± 0.000a	0.408 ± 0.000a	0.436 ± 0.000a	0.437 ± 0.001a
Tartaric acid	0.425 ± 0.006a	0.043 ± 0.001b	0.033 ± 0.002b	0.035 ± 0.001b	0.023 ± 0.000b	0.050 ± 0.001b	-	-	-	-	-	-	-
Fumaric acid	-	-	-	-	-	-	-	-	-	-	-	-	-
Shikimic acid	-	-	-	-	-	-	-	-	-	-	-	-	-

Note: Data are means ± standard deviations of experiments carried out in triplicate. Data in the same row with different letters are significantly different ($p < 0.05$). Samples were collected at 0, 5th, 10th, 15th, 20th, 25th, 30th, 35th, 40th, 45th, 50th, 55th and 60th day, respectively. – means not detected.
